# Anteversion and length of the femoral tunnel in ACL reconstruction: in-vivo comparison between rigid and flexible instrumentation

**DOI:** 10.1186/s40634-019-0198-0

**Published:** 2019-06-22

**Authors:** Frank Wein, Benoit Osemont, Thomas Goetzmann, Adrien Jacquot, Jeremy Valluy, Mo Saffarini, Daniel Molé

**Affiliations:** 1 Centre Artics, Clinique Louis Pasteur, Nancy, France; 2 Radiolor, Clinique Louis Pasteur, Nancy, France; 3 ReSurg S.A, Rue Saint-Jean 22, 1260 Nyon, Switzerland

**Keywords:** ACL, Anteromedial portal, Flexible reamer, Femoral tunnel

## Abstract

**Background:**

Due to it being tangential to the distal femoral axis, the anteromedial portal presents significant risk of causing iatrogenic damage, and of producing tunnels that are too short for optimal osseointegration. Flexible reamers were developed to eliminate the need for knee hyperflexion and offer better-controlled orientation of the femoral tunnel. We aimed to compare the anteversion and length of femoral tunnels drilled using flexible reamers to those drilled using rigid reamers.

**Methods:**

Between May 2012 and December 2013, all patients receiving ACL reconstruction performed by one surgeon were operated on using either a rigid or a flexible reamer from the same supplier (Versi-Tomic® system, Stryker, Kalamazoo, Michigan). The height of each patient was recorded, and the length and anteversion of the femoral tunnels were measured intra-operatively and on true lateral radiographs, respectively.

**Results:**

Thirty-seven patients underwent operations using the rigid instrumentation, and 43 using the flexible instrumentation. There was no statistically significant difference between the two groups in either sex or height (*p* = n.s.). The patients operated on using the rigid instrumentation had tunnels anteverted by 18.6° ± 6° and 33.6 ± 2.9 mm long. Those operated on using the flexible instrumentation had tunnels anteverted by 40° ± 2° and 41.1 ± 3.57 mm long. Both anteversion and tunnel length were significantly greater for tunnels drilled using the flexible instrumentation (*p* < 0.001).

**Conclusions:**

This study demonstrated that flexible reamers produce significantly more anteverted and longer femoral tunnels during ACL reconstruction than rigid reamers. Clinical studies remain necessary to assess the outcomes of ACL reconstruction using flexible reamers.

## Background

Tunnel length and positioning are of crucial importance for the outcomes of anterior cruciate ligament (ACL) reconstruction (Jepsen et al., 2007; Pearle et al., 2015). The superiority of anatomical positioning of the graft and therefore of the aperture of the femoral tunnel has been demonstrated (Hart et al., 2018; Jaecker et al., 2017; Jorge et al., 2018). However, several modern fixation systems require a minimum tunnel length to tightly secure the graft and allow its successful integration (Yamazaki et al., 2006; Zantop et al., 2008a). By allowing more precise positioning and orientation of the femoral tunnel (Kim et al., 2018; Tampere et al., 2018), the anteromedial portal has gained popularity because it facilitates drilling the femoral tunnel independently from the transtibial tunnel and portal (Larson et al., 2012; Lubowitz, 2009; Venosa et al., 2017), which remains associated with a high rate of incorrect femoral tunnel positioning (Dargel et al., 2009; Franceschi et al., 2013).

While there is no consensus on the appropriate length of the femoral tunnel, a number of studies have suggested minimal lengths ranging from 15 mm (Yasuda et al., 2004) to 30 mm (Moon et al., 2014). Due to it being more tangential to the distal femoral axis, the anteromedial portal presents significant risk of producing shorter tunnels, which may compromise successful fixation or osseointegration, and pierce the posterior cortex with the drill, risking damage of posterior tissues such as the peroneal nerve (Hall et al., 2009). In order to avoid these risks, it is common practice to hyperflex the patient’s knee beyond 110° when drilling a femoral tunnel through the anteromedial portal, in order to achieve the required tunnel anteversion. However, this is associated with risks of iatrogenic damage to the cartilage of the medial condyle (Hall et al., 2009; Zantop et al., 2008b) and can prove difficult for patients with bulky or muscular thighs (Lubowitz, 2009). Therefore, even if drilled with the knee at 110° of flexion, femoral tunnels drilled using the anteromedial portal can be too short or insufficiently anteverted (Lubowitz, 2009).

Flexible reamers were developed as a solution to these problems, eliminating the need for knee hyperflexion and offering easier and better-controlled orientation of the femoral tunnel (Alentorn-Geli et al., 2015; Steiner & Smart, 2012). While several cadaveric studies proved the superiority of flexible reamers in ensuring ideal femoral tunnel length and orientation (Steiner & Smart, 2012), few studies evaluated them clinically (Kadija et al., 2017), and none compared both the length and anteversion of the femoral tunnel drilled with rigid versus flexible reamers. The purpose of this study was therefore to evaluate and compare the anteversion and length of femoral tunnels drilled using flexible reamers to those drilled using rigid reamers. The hypothesis was that flexible reamers produce femoral tunnels that are more anteverted and longer than those produced by rigid reamers.

## Methods

Between May 2012 and December 2013, all patients receiving single-bundle ACL reconstruction using patellar tendon autografts performed by the same senior surgeon were operated on using either a rigid or a flexible reamer from the same supplier (Versi-Tomic® system, Stryker, Kalamazoo, Michigan), alternating between the reamers from one operation to another.

### Patient consent

All patients gave written informed consent for their participation in this study, which was approved in advance by the author’s institutional review board (IRB #2019-PM004-FW-001).

### Surgical technique

**Fig. 1:**
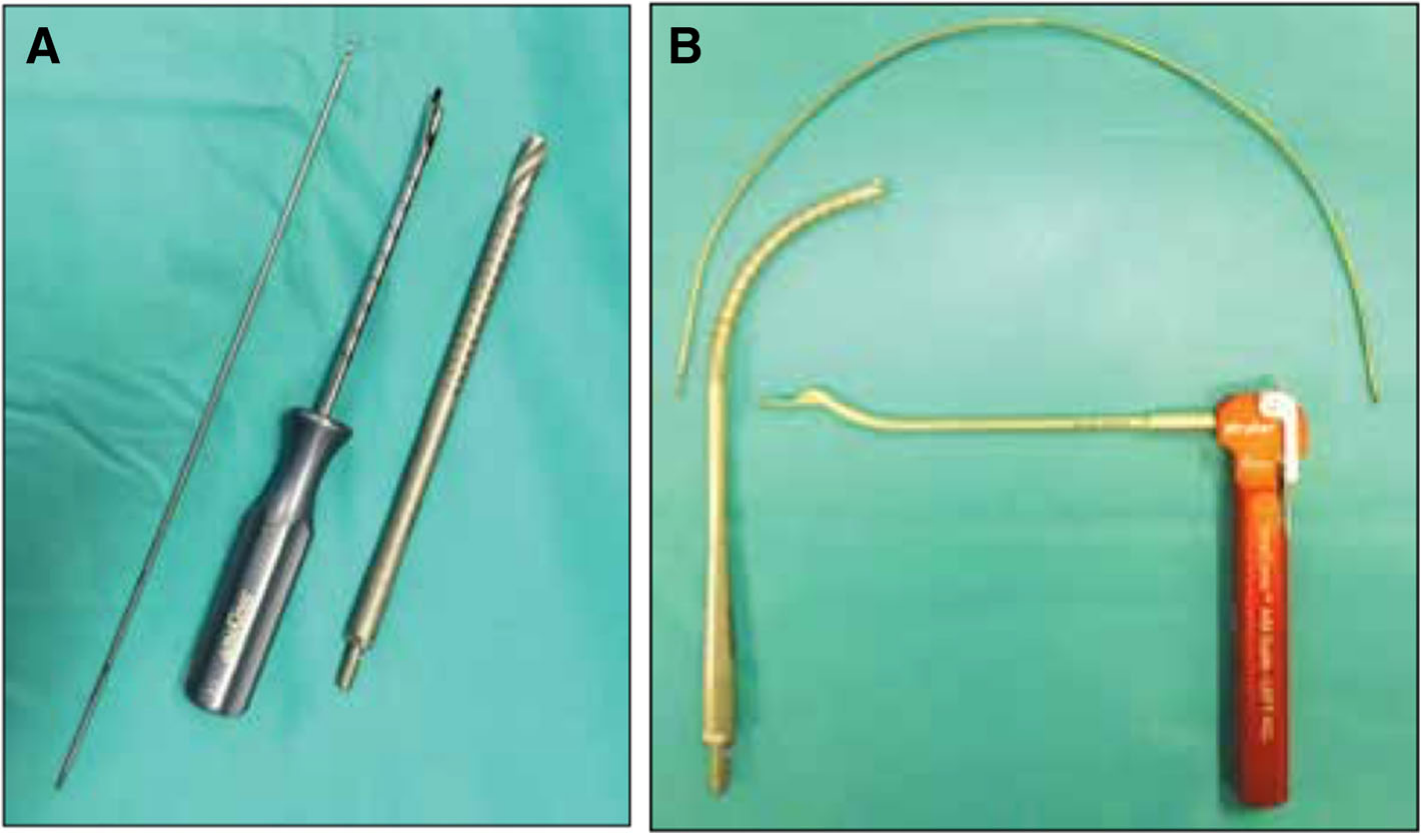
Images of the Versi-Tomic® system with rigid (**a**) and flexible (**b**) reamers

All patients were operated in the supine position, under general anesthesia, using a tourniquet. An arthroscopic investigation confirmed the ligament rupture. All femoral tunnels were drilled blind-ended, from the inside-out, using the anteromedial portal (Lubowitz, 2009), and either rigid or flexible Versi-Tomic® system reamers (Fig. 1). Both rigid and flexible instrumentation used a hooked femoral guide pin placed on the posterior facet of the lateral femoral condyle to ensure correct and standardized tunnel placement. The femoral guide was offset by 6 mm from the articular surface to avoid lateral cortical effraction (Miller et al., 2011). The rigid instrumentation (reamer and pins) required the knees to be flexed at 120°; a rigid guide pin was then inserted into the femur using a femoral guide offset by 6 mm, hooked behind the lateral condyle. A femoral tunnel of diameter 10 mm was then drilled using a rigid reamer following the axis of the guide pin. The flexible instrumentation (reamer and pins) allowed the knee to be flexed only at 90° and also used a femoral guide offset by 6 mm, hooked behind the lateral condyle. The articular extremity of the reamer was anteverted by 42°, thereby angling the flexible pin 42° forward. The flexible reamer then followed the direction imposed by the pin. All patients received patellar tendon autografts fixed with femoral endobuttons and tibial screws.

### Evaluation method

**Fig. 2:**
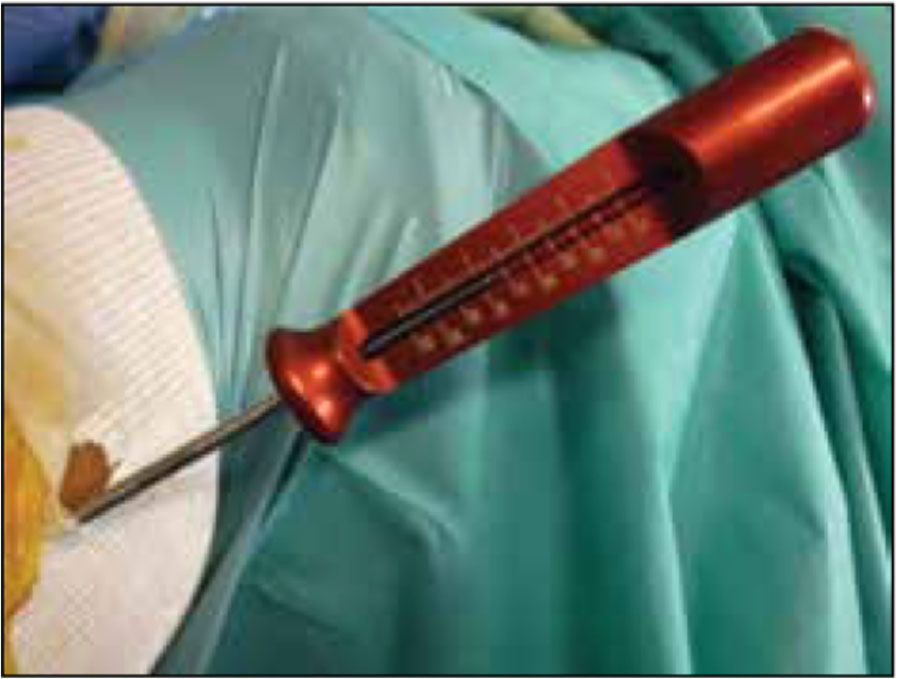
Intraoperative measurement of tunnel length

**Fig. 3:**
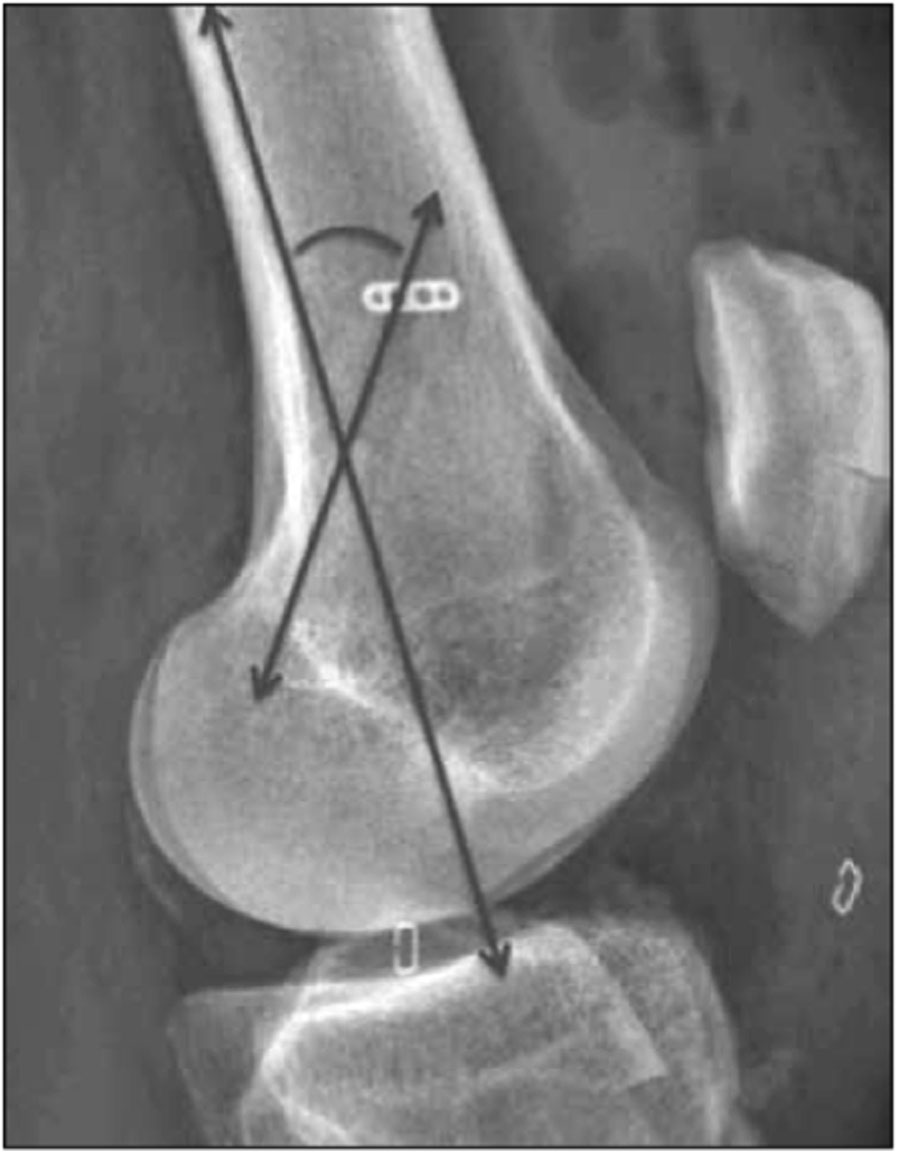
Measurement of femoral tunnel anteversion on true lateral postoperative radiographs. Anteversion is the angle between the posterior cortex of the femoral diaphysis and the line running long the middle of the femoral tunnel ending at the endobutton

The height of the patients was measured during the pre-surgical consultation. The length of the femoral tunnel was measured during the procedure by directly reading the dedicated gauge (Fig. 2). The positioning and anteversion of the femoral tunnels were measured by an independent operator (radiologist, BO) on post-operative true lateral radiographs (Dejour et al., 2017). Anteversion was defined as the angle between the posterior cortex of the femoral diaphysis and the line running along the middle of the femoral tunnel, ending at the endobutton (Fig. 3).

### Statistical analysis

Descriptive statistics were used to summarize the data. Shapiro-Wilk tests were used to assess the normality of distributions. For non-Gaussian quantitative data, differences between groups were evaluated using the Wilcoxon rank sum test (Mann Whitney U test). Statistical analyses were performed using R version 3.3.2 (R Foundation for Statistical Computing, Vienna, Austria). *P*-values < 0.05 were considered statistically significant.

## Results

Thirty-seven patients underwent operations using the rigid instrumentation, of which 23 were men (62%) (Table 1). Their height was 174 ± 8 cm (range, 162–190). Forty-three patients underwent operations using the flexible instrumentation, of which 30 were men (70%). Their height was 176 ± 7 (range, 158–188). There was no statistically significant difference between the two groups in either sex or height (*p* = n.s.).

**Table 1:** Patient demographics

	Rigid Reamer (*n* = 37)		Flexible Reamer (*n* = 43)		*p*-value*
	*mean ± SD*	*(range)*	*mean ± SD*	*(range)*	
Height (cm)	174.1 ± 7.5	(162–190)	175.6 ± 7.0	(158–188)	0.369
Men	23 (62.2%)		30 (69.8%)		0.488

*Wilcoxon rank-sum test or Fisher’s exact test (gender)

The patients operated on using the rigid instrumentation had tunnels anteverted by 19° ± 6° (range, 5°–25°) and 33 ± 3 mm long (range, 30–40) (Table 2). The patients operated on using the flexible instrumentation had tunnels anteverted by 40° ± 2° (range, 35°–45°) and 41 ± 4 mm long (range, 35–50). Both anteversion and tunnel length were significantly greater for tunnels drilled using the flexible instrumentation (*p* < 0.001).

**Table 2:** Tunnel characteristics

	Rigid Reamer (*n* = 37)		Flexible Reamer (*n* = 43)		*p*-value*
	*mean ± SD*	*(range)*	*mean ± SD*	*(range)*	
Length (mm)	33.6 ± 2.9	(30–40)	41.1 ± 3.6	(35–50)	< 0.001
Anteversion (°)	18.6 ± 6.0	(5–25)	40.3 ± 1.7	(35–45)	< 0.001

*Wilcoxon rank-sum test

## Discussion

This study demonstrates that flexible reamers allow the preparation of more anteverted and longer femoral tunnels during ACL reconstruction surgery, without leg hyperflexion. Thus, flexible reamers are likely to reduce the chances of iatrogenic cartilage damage and of graft failure due to insufficient femoral tunnel length. Interestingly, a recent study correlated the angle of the femoral tunnels in the sagittal plane and their length (Wang et al., 2017) when using the transportal technique. It is possible that this is also true for the anteromedial portal and therefore that the greater length observed when drilling femoral tunnels with flexible reamers is in part due to their greater anteversion. It follows that the adaptability of flexible reamers to drill at optimal angles could be key to the success of the anteromedial portal by providing improved safety and adequate tunnel length.

In this study, we found that the use of flexible reamers also allowed markedly greater anteversion than with rigid reamers with  flexing the legs to 120° (mean anteversion, 40° ± 2° vs 19° ± 6°). These anteverted tunnels end on the lateral surface of the femur, away from the articular cartilage or posterior tissues, and avoid the risk of cartilage damage associated with the anteromedial portal (Farrow & Liu, 2010; Hall et al., 2009; Nakamura et al., 2009). As such, the use of flexible reamers would eliminate the requirement for knee hyperflexion, which is associated with iatrogenic risks to the cartilage or posterior structures (Hall et al., 2009; Zantop et al., 2008b).

This study also found that flexible reamers granted longer tunnels than rigid reamers (41 ± 4 mm vs 34 ± 3 mm). This finding is consistent with previous cadaver studies (Silver et al., 2010; Steiner & Smart, 2012) and clinical studies (Kadija et al., 2017). While there is no consensus on the appropriate tunnel length for optimal graft osseointegration, a number of studies suggested minimal tunnel lengths ranging from 15 mm (Yasuda et al., 2004) to 30 mm (Moon et al., 2014), although the minimal length using suspensory fixations could be even longer due to the extra space required by the suspension system. Therefore, the range of tunnels obtained with a rigid reamer in this study of 30 to 40 mm (mean, 34 ± 3 mm) is likely to be insufficient for suspensory fixation in several cases. With a range of 35 to 50 mm (mean, 41 ± 4), the tunnels obtained with the flexible reamer are more likely to provide adequate length for optimal osseointegration using suspensory fixations. Our in-vivo findings corroborate those of a previous cadaveric study (Steiner & Smart, 2012), which suggested that flexible reamers produced longer tunnels than rigid reamers, and refute the findings of another cadaveric study that suggested the contrary (Larson et al., 2012).

This study brings together and re-evaluates previous findings using a controlled protocol: both study groups are comparable in number, size and gender, and are operated by the same surgeon using the same instrumentation with either rigid or flexible Versi-Tomic® system reamers. Furthermore, this study is, to the authors’ knowledge, the first to evaluate tunnel anteversion on true lateral radiographs. Taken together, these findings suggest that flexible reamers may represent an elegant solution to the pitfalls of the anteromedial portal (Lubowitz, 2009). However, this study has limitations. Firstly, tunnel positioning was not evaluated, so it is not possible to assert that both reamers in this controlled clinical study provide equivalent anatomical positioning. Secondly, tunnel orientation in the frontal plane was not recorded, and our radiographic measurements are less accurate than computed tomography measurements, which were not acquired to avoid exposing patients to radiation. Finally, with no clinical follow-up the superiority of flexible reamers over rigid reamers could not be demonstrated.

This study revealed that flexible reamers produce more anteverted and longer femoral tunnels during ACL reconstruction. As a result, they are likely to provide reduced iatrogenic risks, more optimal graft osseointegration, and an elegant solution to the pitfalls of the anteromedial portal. Clinical studies remain necessary to assess the outcomes of ACL reconstruction using flexible reamers.

## Abbreviation

ACL: Anterior Cruciate Ligament

## References

[CR1] Alentorn-Geli E, Stuart JJ, Choi JH, Toth AP, Moorman CT, Taylor DC (2015). Inside-out Antegrade Tibial tunnel drilling through the posterolateral portal using a flexible reamer in posterior cruciate ligament reconstruction. Arthrosc Tech.

[CR2] Dargel J, Schmidt-Wiethoff R, Fischer S, Mader K, Koebke J, Schneider T (2009). Femoral bone tunnel placement using the transtibial tunnel or the anteromedial portal in ACL reconstruction: a radiographic evaluation. Knee Surg Sports Traumatol Arthrosc.

[CR3] Dejour D, La Barbera G, Pasqualotto S, Valoroso M, Nover L, Reynolds R, Saffarini M (2017). Sagittal plane corrections around the knee. J Knee Surg.

[CR4] Farrow LD, Liu RW (2010). Lateral anatomic structures at risk during transepiphyseal anterior cruciate ligament reconstruction. J Knee Surg.

[CR5] Franceschi F, Papalia R, Rizzello G, Del Buono A, Maffulli N, Denaro V (2013). Anteromedial portal versus transtibial drilling techniques in anterior cruciate ligament reconstruction: any clinical relevance? A retrospective comparative study. Arthroscopy.

[CR6] Hall MP, Ryzewicz M, Walsh PJ, Sherman OH (2009). Risk of iatrogenic injury to the peroneal nerve during posterolateral femoral tunnel placement in double-bundle anterior cruciate ligament reconstruction. Am J Sports Med.

[CR7] Hart A, Sivakumaran T, Burman M, Powell T, Martineau PA (2018). A prospective evaluation of femoral tunnel placement for anatomic anterior cruciate ligament reconstruction using 3-dimensional magnetic resonance imaging. Am J Sports Med.

[CR8] Jaecker V, Zapf T, Naendrup JH, Pfeiffer T, Kanakamedala AC, Wafaisade A, Shafizadeh S (2017). High non-anatomic tunnel position rates in ACL reconstruction failure using both transtibial and anteromedial tunnel drilling techniques. Arch Orthop Trauma Surg.

[CR9] Jepsen CF, Lundberg-Jensen AK, Faunoe P (2007). Does the position of the femoral tunnel affect the laxity or clinical outcome of the anterior cruciate ligament-reconstructed knee? A clinical, prospective, randomized, double-blind study. Arthroscopy.

[CR10] Jorge PB, Escudeiro D, Severino NR, Santili C, de Paula Leite Cury R, Junior AD, Guglielmetti LGB (2018). Positioning of the femoral tunnel in anterior cruciate ligament reconstruction: functional anatomical reconstruction. BMJ Open Sport Exerc Med.

[CR11] Kadija M, Milovanovic D, Bumbasirevic M, Carevic Z, Dubljanin-Raspopovic E, Stijak L (2017). Length of the femoral tunnel in anatomic ACL reconstruction: comparison of three techniques. Knee Surg Sports Traumatol Arthrosc.

[CR12] Kim SH, Kim SJ, Choi CH, Kim D, Jung M (2018). Optimal condition to create femoral tunnel considering combined influence of knee flexion and transverse drill angle in anatomical single-bundle ACL reconstruction using medial portal technique: 3D simulation study. Biomed Res Int.

[CR13] Larson AI, Bullock DP, Pevny T (2012). Comparison of 4 femoral tunnel drilling techniques in anterior cruciate ligament reconstruction. Arthroscopy.

[CR14] Lubowitz JH (2009). Anteromedial portal technique for the anterior cruciate ligament femoral socket: pitfalls and solutions. Arthroscopy.

[CR15] Miller CD, Gerdeman AC, Hart JM, Bennett CG, Golish SR, Gaskin C, Miller MD (2011). A comparison of 2 drilling techniques on the femoral tunnel for anterior cruciate ligament reconstruction. Arthroscopy.

[CR16] Moon DK, Yoon CH, Park JS, Kang BJ, Cho SH, Jo HS, Hwang SC (2014). Effect of anteromedial portal entrance drilling angle during anterior cruciate ligament reconstruction: a three-dimensional computer simulation. Yonsei Med J.

[CR17] Nakamura M, Deie M, Shibuya H, Nakamae A, Adachi N, Aoyama H, Ochi M (2009). Potential risks of femoral tunnel drilling through the far anteromedial portal: a cadaveric study. Arthroscopy.

[CR18] Pearle AD, McAllister D, Howell SM (2015). Rationale for strategic graft placement in anterior cruciate ligament reconstruction: I.D.E.a.L. femoral tunnel position. Am J Orthop (Belle Mead NJ).

[CR19] Silver AG, Kaar SG, Grisell MK, Reagan JM, Farrow LD (2010). Comparison between rigid and flexible systems for drilling the femoral tunnel through an anteromedial portal in anterior cruciate ligament reconstruction. Arthroscopy.

[CR20] Steiner ME, Smart LR (2012). Flexible instruments outperform rigid instruments to place anatomic anterior cruciate ligament femoral tunnels without hyperflexion. Arthroscopy.

[CR21] Tampere T, Devriendt W, Cromheecke M, Luyckx T, Verstraete M, Victor J (2018) Tunnel placement in ACL reconstruction surgery: smaller inter-tunnel angles and higher peak forces at the femoral tunnel using anteromedial portal femoral drilling-a 3D and finite element analysis. Knee Surg Sports Traumatol Arthrosc. 10.1007/s00167-018-5272-0. 10.1007/s00167-018-5272-0 30406406

[CR22] Venosa M, Delcogliano M, Padua R, Alviti F, Delcogliano A (2017). Femoral tunnel positioning in anterior cruciate ligament reconstruction: anteromedial portal versus Transtibial technique-a randomized clinical trial. Joints.

[CR23] Wang JH, Lee DK, Chung ST, Lee BH (2017). Influence of change of tunnel axis angle on tunnel length during double-bundle ACL reconstruction via the transportal technique. BMC Musculoskelet Disord.

[CR24] Yamazaki S, Yasuda K, Tomita F, Minami A, Tohyama H (2006). The effect of intraosseous graft length on tendon-bone healing in anterior cruciate ligament reconstruction using flexor tendon. Knee Surg Sports Traumatol Arthrosc.

[CR25] Yasuda K, Kondo E, Ichiyama H, Kitamura N, Tanabe Y, Tohyama H, Minami A (2004). Anatomic reconstruction of the anteromedial and posterolateral bundles of the anterior cruciate ligament using hamstring tendon grafts. Arthroscopy.

[CR26] Zantop T, Ferretti M, Bell KM, Brucker PU, Gilbertson L, Fu FH (2008). Effect of tunnel-graft length on the biomechanics of anterior cruciate ligament-reconstructed knees: intra-articular study in a goat model. Am J Sports Med.

[CR27] Zantop T, Haase AK, Fu FH, Petersen W (2008). Potential risk of cartilage damage in double bundle ACL reconstruction: impact of knee flexion angle and portal location on the femoral PL bundle tunnel. Arch Orthop Trauma Surg.

